# Real-Time Digitized Visual Feedback in Exercise Therapy for Lower Extremity Functional Deficits: Qualitative Study of Usability Factors During Prototype Testing

**DOI:** 10.2196/51771

**Published:** 2024-12-10

**Authors:** Klaus Widhalm, Lukas Maul, Sebastian Durstberger, Peter Putz, Carissa Klupper, Franz Werner

**Affiliations:** 1 Physiotherapy FH Campus Wien, University of Applied Sciences Vienna Austria; 2 Institute for Biomechanics Paracelsus Medical University Salzburg Austria; 3 Reseach Center Health Sciences FH Campus Wien, University of Applied Sciences Vienna Austria; 4 Research Center Digital Health and Care FH Campus Wien, University of Applied Sciences Vienna Austria; 5 Health Assisting Engineering FH Campus Wien, University of Applied Sciences Vienna Austria

**Keywords:** visualization, lower extremity, digitized visual feedback, exercise therapy, functional deficit, serious game, rehabilitation, osteoarthritis, usability, physiotherapy, mobile phone

## Abstract

**Background:**

Osteoarthritis is one of the most common degenerative diseases of the musculoskeletal system and can ultimately lead to the need for surgery, such as total knee or hip arthroplasty. Functional movement deficits can be a prognostic factor for osteoarthritis in the lower extremities. Thus, training physiological movement patterns may help in the treatment of such functional deficits. Motivation to exercise frequently is of utmost importance and can be promoted by using digital real-time feedback.

**Objective:**

This qualitative study aims to gather user recommendations for prototype feedback visualizations in a real-time exercise-feedback system called homeSETT for the treatment of functional deficits. The system provides real-time feedback to participants while performing exercises that focus on functional deficits, such as lateral trunk lean, pelvic drop, and valgus thrust. The findings of this study should help to optimize the prototype feedback visualizations. Thus, the main research questions were how patients, physiotherapists, and physicians evaluate the presented, current state of prototype feedback visualizations for selected functional exercises, and what improvements and variations would be recommended.

**Methods:**

Testing of the prototype feedback visualizations took place at a movement laboratory using a 3D optoelectronic movement analysis system. Data on usability factors were acquired using the thinking aloud method during and semistructured interviews after prototype testing. Transcribed audio recordings of semistructured interviews as well as scribing logs of the thinking aloud method were examined using qualitative content analysis.

**Results:**

Data were analyzed from 9 participants, comprising 2 (22%) patients, 2 (22%) physicians, and 5 (56%) physiotherapists. The mean age of the participants was 45 (SD 9) years and the mean work experience among the participating physiotherapists and physicians was 22 (SD 5) years. Each participant tested 11 different exercise-feedback combinations. Overall, results indicated that participants enjoyed the prototype feedback visualizations and believed that they could be used in therapeutic settings. Participants appreciated the simplicity, clarity, and self-explanatory nature of the feedback visualizations. While most participants quickly familiarized themselves, some struggled to recognize the feedback goals and connect the visualizations to their movements. Recommendations for improvement included optimizing color schemes, sensitivity, and difficulty adjustments. Adding instructional information and game design elements, such as repetition counting and reward systems, was deemed useful. The main study limitations were the small sample size and the use of feedback on performance as the sole feedback modality.

**Conclusions:**

The prototype feedback visualizations were positively perceived by the participants and were considered applicable in therapy settings. Insights were gathered on improving the color scheme, sensitivity, and recognizability of the feedback visualizations. The implementation of additional gamification and instructional elements was emphasized. Future work will optimize the prototype feedback visualizations based on study results and evaluate the homeSETT system’s efficacy in eligible patient populations.

## Introduction

### Background

Degenerative musculoskeletal conditions are increasingly burdening both patients and public health care systems, driven by demographic changes and rising costs [1-4]. Among these conditions, osteoarthritis is particularly prevalent, affecting >40 million individuals in Europe [5]. Globally, this results in >18 million years lived annually with the disease. Degenerative conditions, such as osteoarthritis of the lower extremities, often necessitate total knee or hip arthroplasty [6].

Research indicates that physical inactivity can lead to osteoarthritis [7] and dysfunctional movement patterns, such as lateral trunk lean, can be predictors for osteoarthritis in the lower extremities [8,9]. Addressing these functional deficits and promoting the relearning of physiologically favorable movement patterns are key to preventing and rehabilitating osteoarthritis, as well as aiding recovery following knee or hip arthroplasty. However, patients often lack sufficient motivation to engage in regular, autonomous physical activity even though exercise and effective repetition of correct movement patterns are essential for achieving desired learning effects [10-14]. A lack of motivation can adversely affect the learning process, primarily due to 2 factors—a general lack of interest in exercise [15] and conflicts with other daily life interests and responsibilities [16].

Motivation to exercise can be positively enhanced through real-time feedback incorporating serious gaming elements, among other supportive methods [17]. The integration of digital feedback mechanisms in exercise games has the potential to be engaging, enjoyable, and thus, a significant source of motivation for participants or patients. Various gamification strategies have been highlighted for their ability to improve therapy adherence and encourage patient participation [18]. Moreover, visual feedback control can substantially enhance learning effects. Zemková and Hamar [19] demonstrated that task-oriented, sensorimotor exercises are performed considerably better with visual feedback control than without visual feedback control.

### Objectives

This qualitative study aimed to evaluate usability factors of prototype on-screen visualizations of a real-time visual feedback system for exercise therapy targeting neuromuscular functional deficits such as lateral trunk lean, pelvic drop, and valgus thrust. The prototype visualizations were tested on the Gait Real-Time Analysis Laboratory (GRAIL) system (Motek Medical BV). Following this evaluation, the visualizations will be incorporated into the prototype homeSETT, a portable marker-less exercise-feedback device developed in the research project SETT (Smart Real-Time Feedback Assisted Exercise Therapy). Patients, physiotherapists, and physicians were eligible to participate in this qualitative study to generate broad feedback on the prototype feedback visualizations. The knowledge gained should highlight usability issues and improve the understanding of individually required visualizations of real-time feedback for exercise therapy, targeting the listed functional deficits for prevention and rehabilitation. These findings will be used to optimize the prototype feedback visualizations according to the needs and suggestions expressed by patients, physiotherapists, and physicians. This should ensure that the prototype feedback visualizations within the homeSETT system are accepted by all end users and are applicable within a therapeutic process.

The development of the prototype feedback visualizations as well as the whole homeSETT system is based on the human-centered design approach. According to International Organization for Standardization 9241-210:2010(E) [20], this is used to focus on users’ needs and requirements for interactive systems and aims to make systems usable and useful. Through the findings of this study, we hope to gain a better understanding of the individual visualizations needed to provide real-time feedback for exercises that target functional deficits. Therefore, 2 main research questions were pursued: How do patients, physiotherapists, and physicians evaluate the presented state of real-time visual feedback for selected functional exercises regarding usability factors and acceptance? What improvements and variations can be made to the visual feedback displayed?

## Methods

### Study Design

We present a qualitative study examining usability factors of prototype feedback visualizations for real-time exercise feedback. We used 2 qualitative methods for data acquisition and a content structuring approach for data analysis. The thinking aloud method proposed by Ericsson and Simon [21] in combination with scribing logs presented by Eaton et al [22] were used to collect field data during the prototype testing. After prototype testing semistructured interviews based on the approach of Meuser and Nagel [23] were conducted to gather in-depth information about the participants’ views of the prototype feedback visualizations for real-time exercise feedback. In addition, a questionnaire was used to gather participant-reported information on technical affinity before prototype testing. The Consolidated Criteria for Reporting Qualitative Research (COREQ) checklist [24] was used to report the study findings (Multimedia Appendix 1).

### Sampling Strategy

Data from previous studies showed that usability testing using the thinking aloud method does not require a large sample size to successfully identify usability problems. It has been shown that up to 85% saturation in the identification of usability problems can be achieved with >5 participants [25]. Therefore, a goal of including 10 participants in this study was set to achieve saturation regarding the reporting of usability problems. The prototype feedback visualizations were iterated several times before the study, based on the results of internal expert workshops; focus groups; and workshops with physicians, physiotherapists, and patients as summarized in a previous publication by Widhalm et al [26]. The results of this study represent the final iteration process for the design of the on-screen prototype feedback visualizations. Details on the development of the prototype feedback visualizations are available in Multimedia Appendix 2. Participants were recruited using nonprobability, purposive sampling according to eligibility criteria for the prototype testing [27]. Participants were recruited by contacting several networks of the FH Campus Wien University of Applied Sciences, Vienna (FHCW) via email in September 2022. The FHCW physiotherapy staff was contacted via email with the study information. In addition, physiotherapists and physicians outside the FHCW were informed about the study via email and publicly available contact information. Patients were recruited through personal contact with the help of publicly available contact information, outpatient rehabilitation centers, and physicians at the Orthopedic Hospital Speising (OSS).

### Inclusion and Exclusion Criteria

Participants aged between 18 and 65 years who belonged to 1 of the 3 (physiotherapists, patients, and physicians) user groups and who met their specific inclusion criteria were included in the study.

Physiotherapists who (1) were actively working at the time of study participation, and (2) had at least 2 years of professional experience in the field of orthopedic physical therapy after obtaining professional certification (intramural and extramural professional experience were considered equally) were included in this study.

Patients who (1) were currently or previously experiencing lower extremity musculoskeletal conditions, such as osteoarthritis, and (2) had undergone surgery at least 3 months before, if they had already undergone any arthroplastic or reconstructive surgery, were included in this study.

Physicians with at least 2 years of professional experience in the field of orthopedics or rehabilitation were included in this study.

Individuals in any of these groups were excluded if (1) they had acute pain or inflammation, and (2) the performed health and flexibility check indicated that they could not participate in the study due to functional or physiological limitations.

### Ethical Considerations

The study was conducted in accordance with the principles of the Declaration of Helsinki. Ethics approval for the study protocol was obtained from the FHCW Ethics Commission (ethics commission number 62/2022). The principal investigator instructed the participants, explained the expected benefits and risks of the study, and answered open questions from the participants. Written informed consent was obtained from all participants. Shopping vouchers were given out as compensation for about 2 hours of study participation. No other incentives were offered. All participants were covered by clinical trial insurance for all study-related procedures.

### Data Collection

The prototype testing took place in the movement laboratory of the FHCW. Feedback testing was performed on the GRAIL system, which consists of a dual belt instrumented treadmill, a 3D optoelectronic motion analysis system (Vicon Motion Systems Limited), and a 180° circular projection screen. Prototype feedback visualizations were run in D-Flow software (Motek Medical BV) and projected onto the screen. Parameters presented in the feedback visualization were processed in real time from marker coordinates, captured by the motion analysis system. The GRAIL system was chosen for developing and testing the prototype feedback visualizations due to the versatility of the D-Flow software, which allows for rapid and straightforward prototyping.

All participants were first verbally informed of the procedures and methods by the principal investigator. After participants’ questions were sufficiently answered, informed consent was obtained. Appropriate clothing was provided as needed. Demographic data and self-reported technical affinity data were collected. A health and flexibility assessment was conducted, consisting of a structured interview about the presence of musculoskeletal conditions and a test of active flexibility. Participants then practiced the thinking aloud method during the application of 26 retroreflective markers on bony landmarks. Participants were guided onto the treadmill, a safety harness was fastened by a member of the study team, and a calibration trial was recorded. Using a harness as safety measures was implemented due to the elevated position of the treadmill and is not necessary when the homeSETT system is used on level ground. Audio and video recordings were then started and participants evaluated the prototype feedback visualization using the thinking aloud method while performing 7 different functional exercises, namely squat, squat-lunge, lunge, single-limb squat, step-up and step-down, and single-limb stance pelvic drop. Each exercise was performed with the associated feedback visualization for up to 5 minutes per exercise. Each feedback visualization was related to a body region which could be either right or left knee, hip and pelvis, or trunk. All feedback visualizations focused on 1 of the 3 deviations—lateral trunk lean, pelvic drop, and valgus thrust. Therefore, the feedback visualizations can be categorized as concurrent and kinematic and the feedback modality can be described as knowledge of performance [28]. Overall, each participant tested the feedback visualizations for 11 different exercise-feedback combinations. Feedback visualizations exercises 1 through 5.2 indicated whether participants maintained stability in the targeted body region during exercise or if any of the 3 types of deviations occurred. Exercises 6.1 and 6.2 were reward-based visualizations that included a shooting-star animation when the participants could execute the exercise within a certain range of motion. An overview of the exercises performed in combination with the corresponding prototype feedback visualization is presented in Table 1.

**Figure 1:**
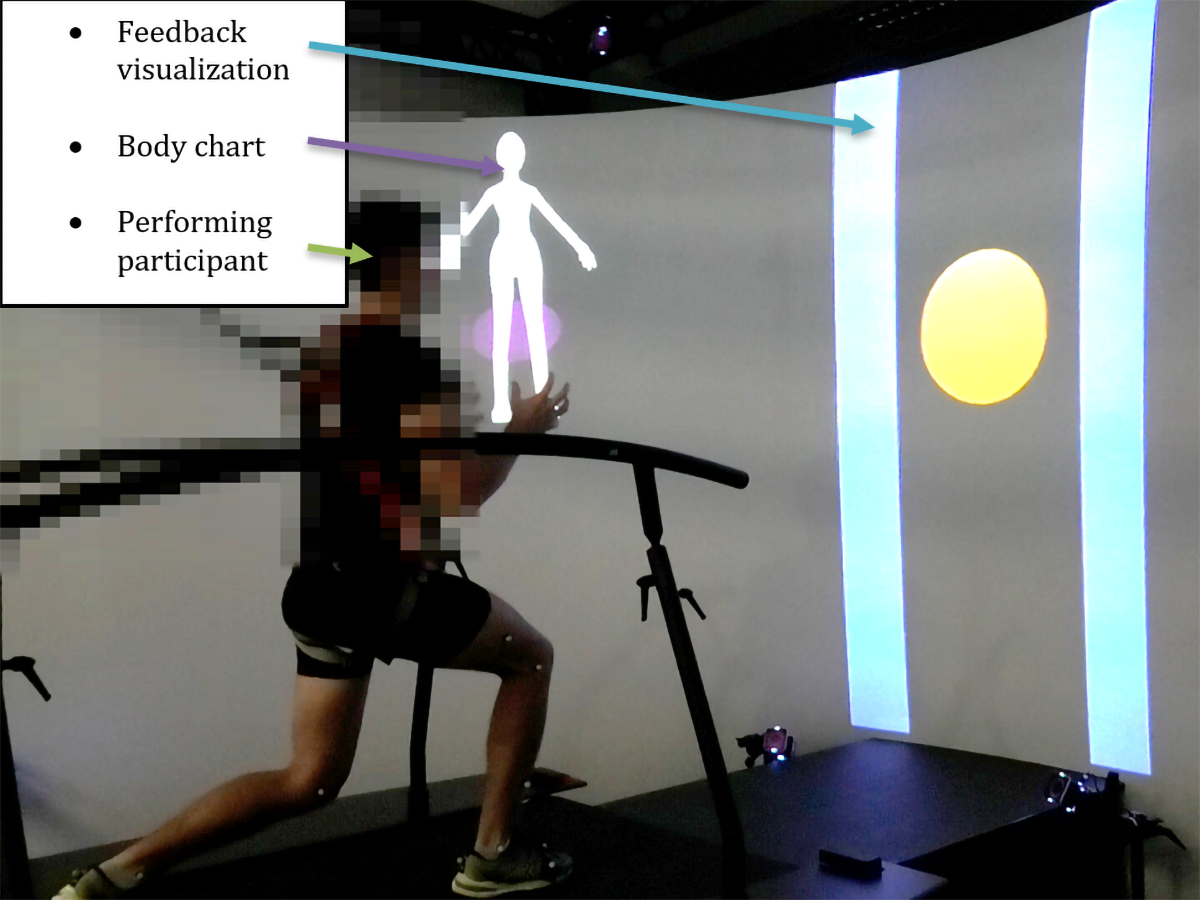
Feedback visualization of Exercise 2.1 on the Gait Real-Time Analysis Laboratory System showing the setup and different feedback elements.

Participants were guided through the prototype testing by a physiotherapist to ensure participant safety and correct exercise execution. The physiotherapist was allowed to ask open-ended questions if the thinking aloud process was not initiated by the participants or to keep the thinking aloud process going by encouraging the participants to keep talking while testing. The prototype testing was accompanied by a second researcher taking the scribing logs and a third researcher operating the GRAIL system. After all exercises and feedback visualizations had been tested, the participants stepped off the treadmill, the markers and harness were removed, and the participants were enquired about their wellbeing. The data recording phase was followed by a face-to-face semistructured interview. The duration of all study-related procedures was a maximum of 2 hours per participant. Semistructured interviews lasted from 6 to 19 minutes. Figure 1 shows the system setup for testing the prototype feedback visualizations on the GRAIL system. Videos of the system setup and testing of the prototype feedback visualizations for exercises 1 to 6.2 on the GRAIL system are available in Multimedia Appendix 3.

**Table 1:** Overview of the feedback visualizations and corresponding exercises.

Feedback exercise number	Feedback name	Exercise	Body region highlighted in body chart	Deviation	Feedback and body chart visualization
Exercise 1^a^	Vertical tacho	Squat	Left knee	Valgus thrust	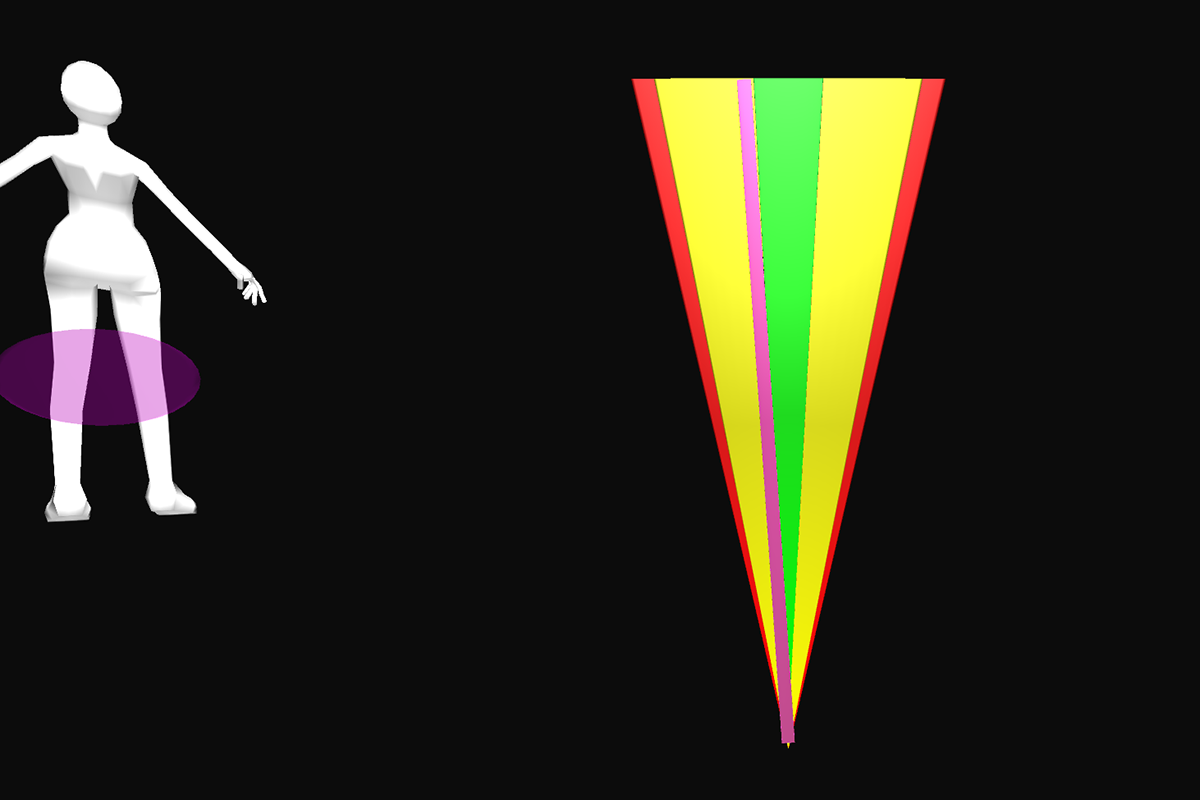
Exercise 2.1	Ball	Squat-lunge	Right knee	Valgus thrust	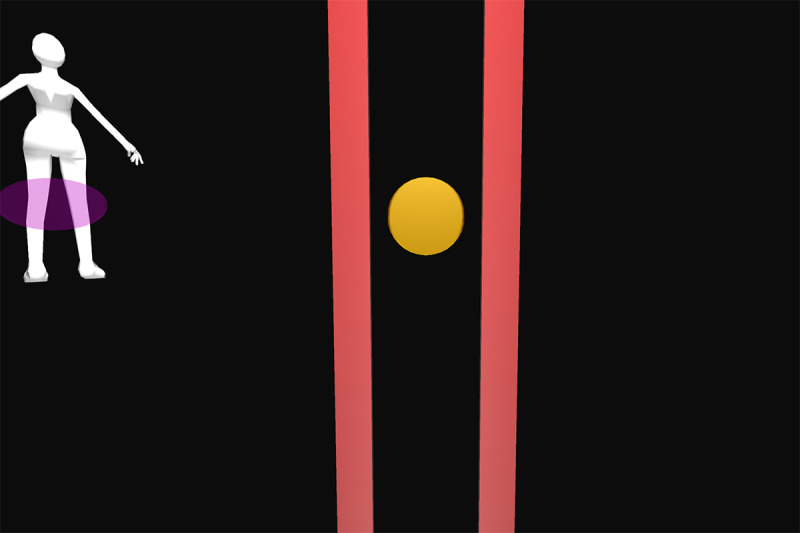
Exercise 2.2	Body model	Squat-lunge	Trunk	Trunk lean	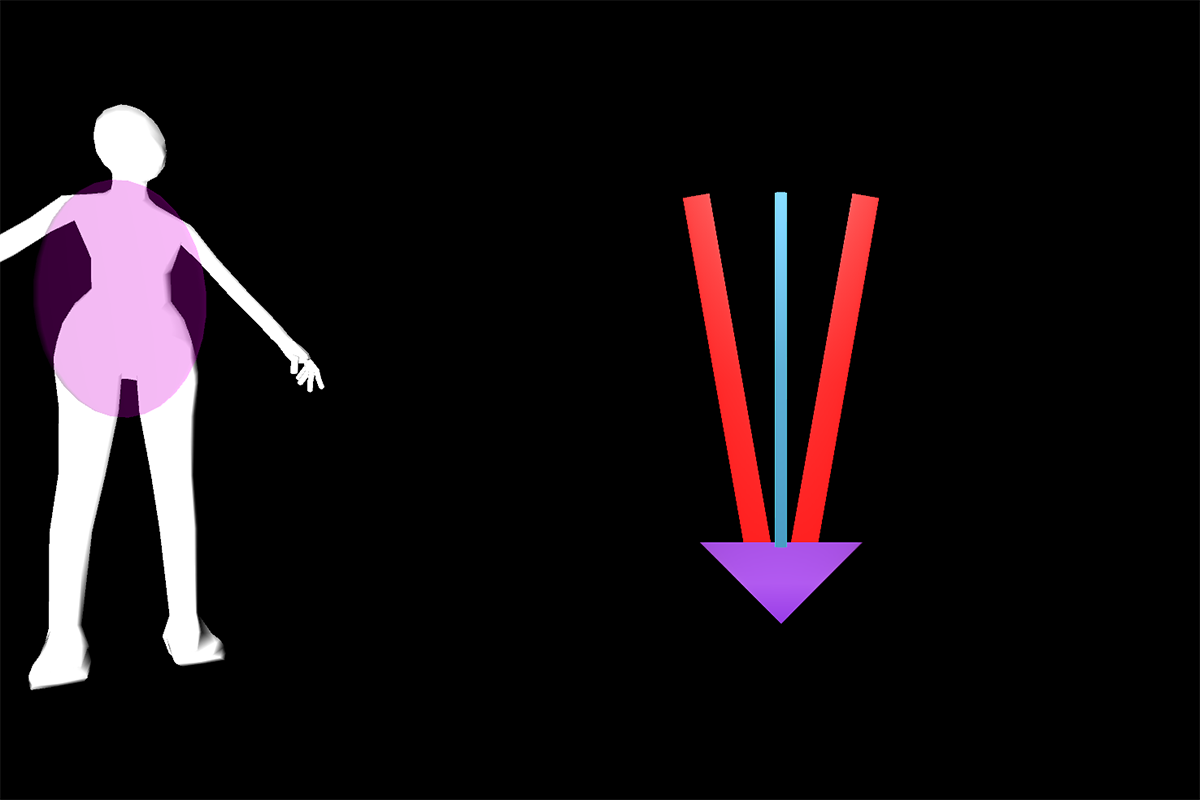
Exercise 3.1	Single bar	Lunge	Hip and pelvis	Pelvic drop	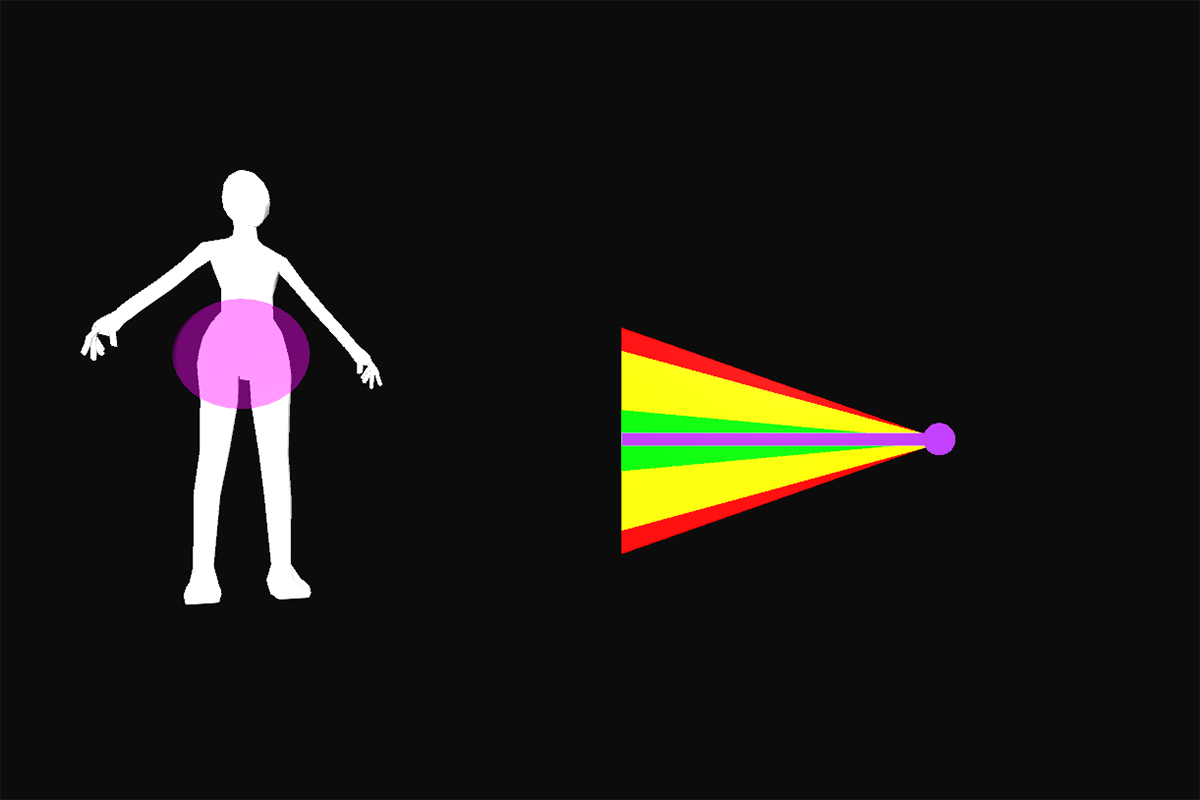
Exercise 3.2	Vertical tacho	Lunge	Left knee	Valgus thrust	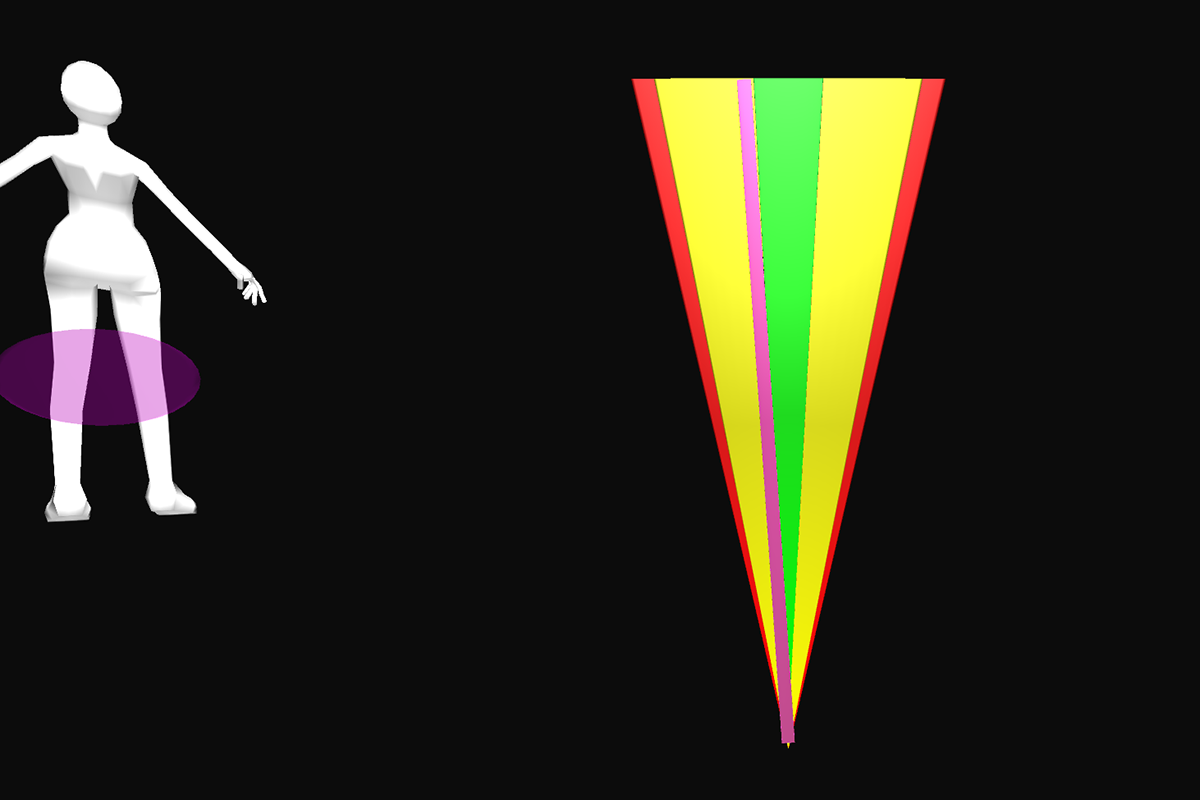
Exercise 4.1	Ball	Single-limb squat	Right knee	Valgus thrust	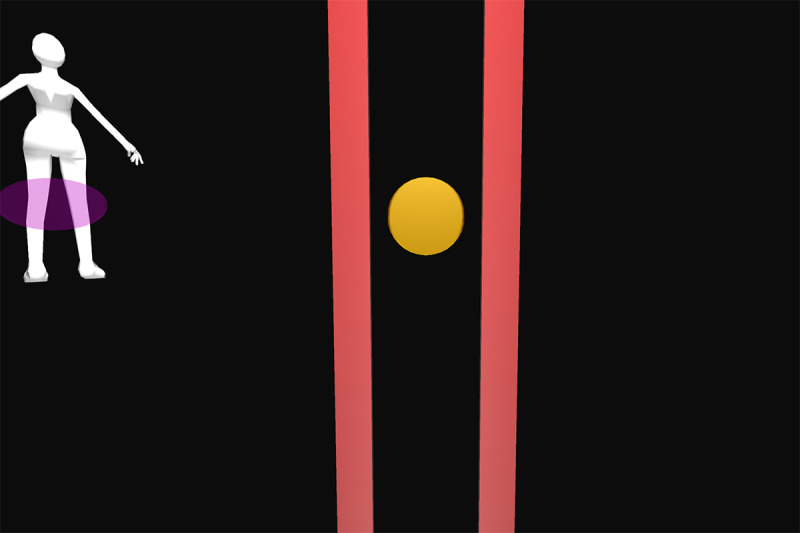
Exercise 4.2	Horizontal tacho	Single-limb squat	Hip and pelvis	Pelvic drop	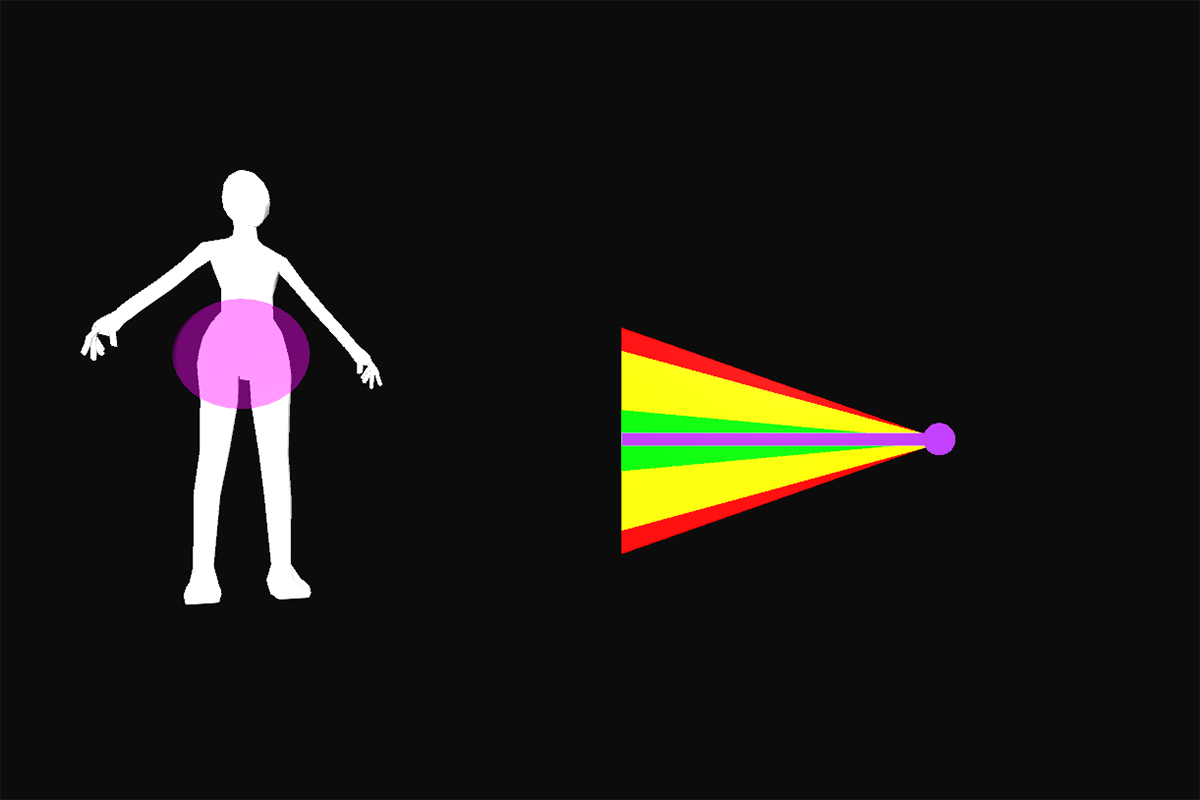
Exercise 5.1	Bar	Step up	Left knee	Valgus thrust	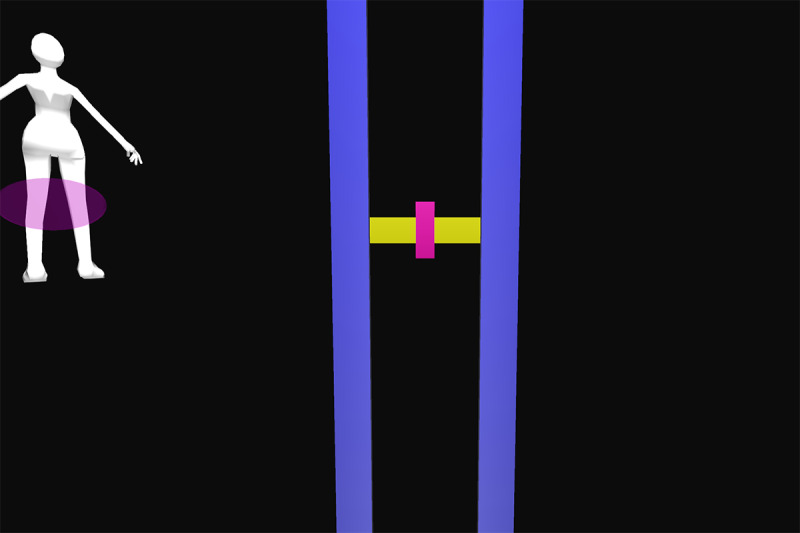
Exercise 5.2	Horizontal bar	Step up	Hip and pelvis	Trunk lean	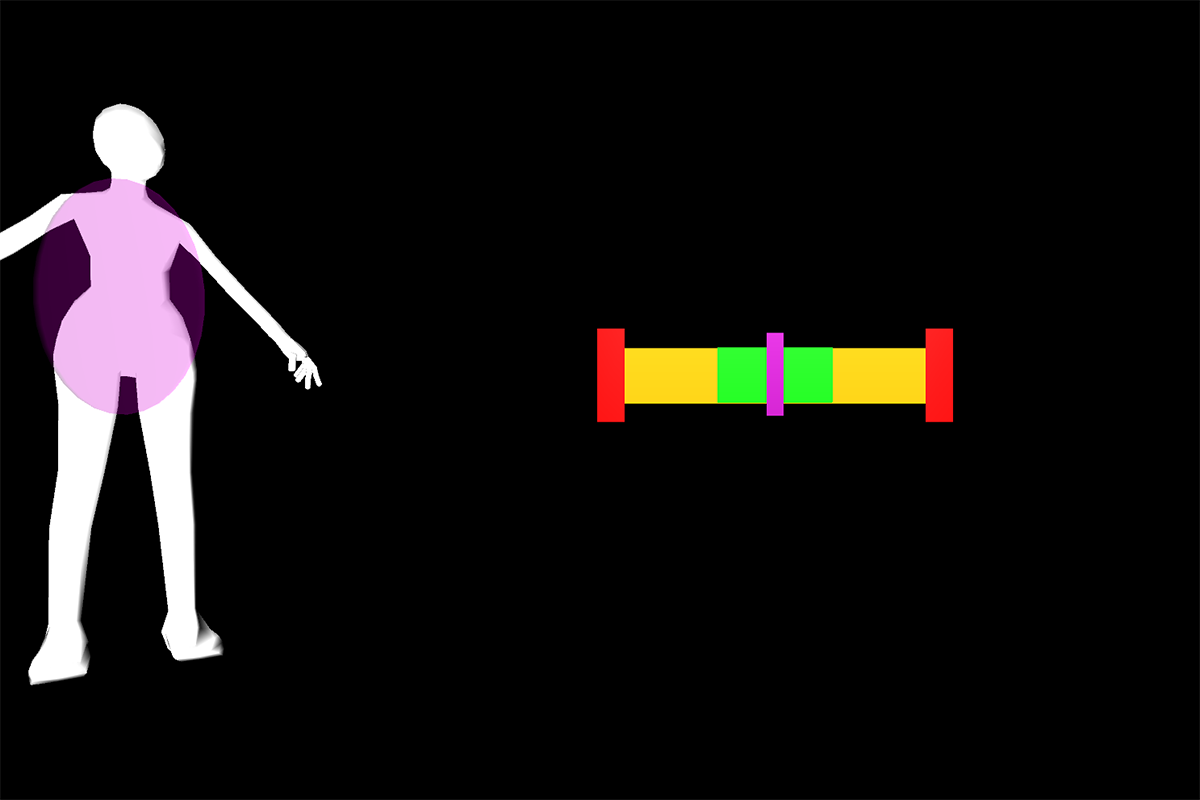
Exercise 6.1	Reward tacho	Single-limb stance pelvic drop and lift	Hip and pelvis	Pelvic drop and lift	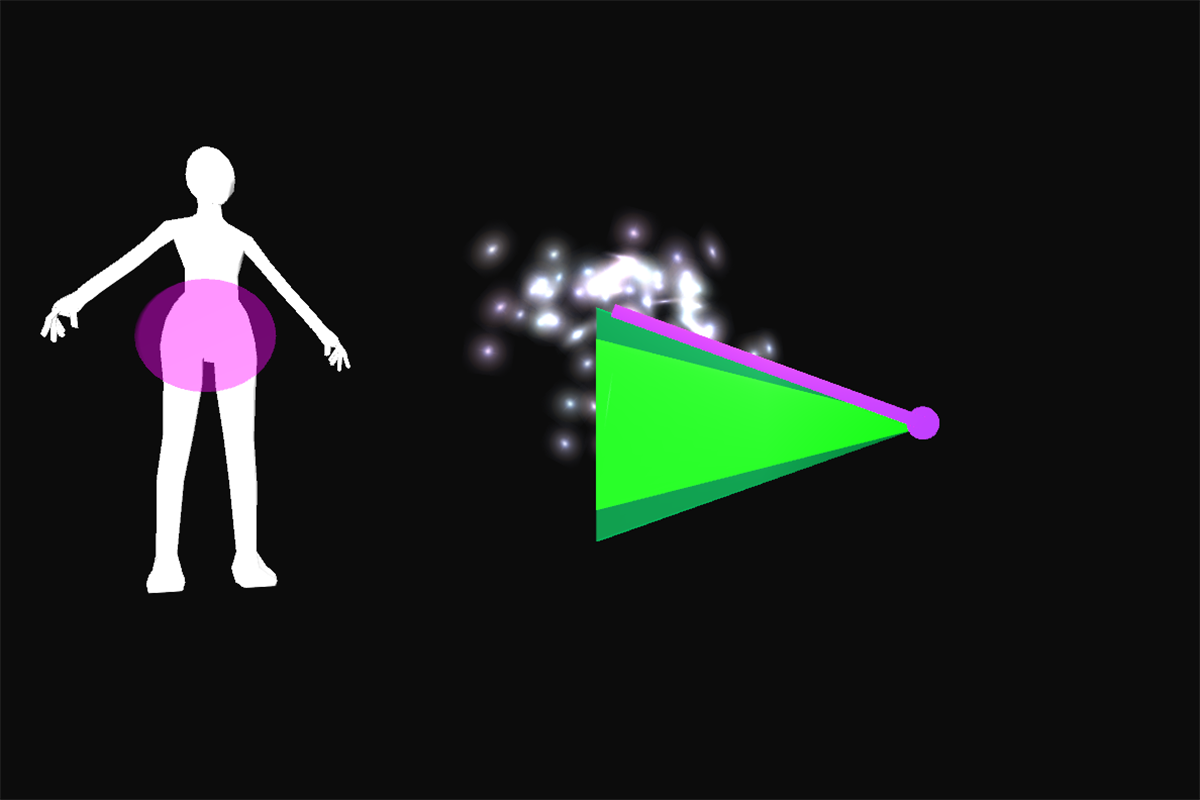
Exercise 6.2	Reward flying bar	Single-limb stance pelvic drop and lift	Hip and pelvis	Pelvic drop and lift	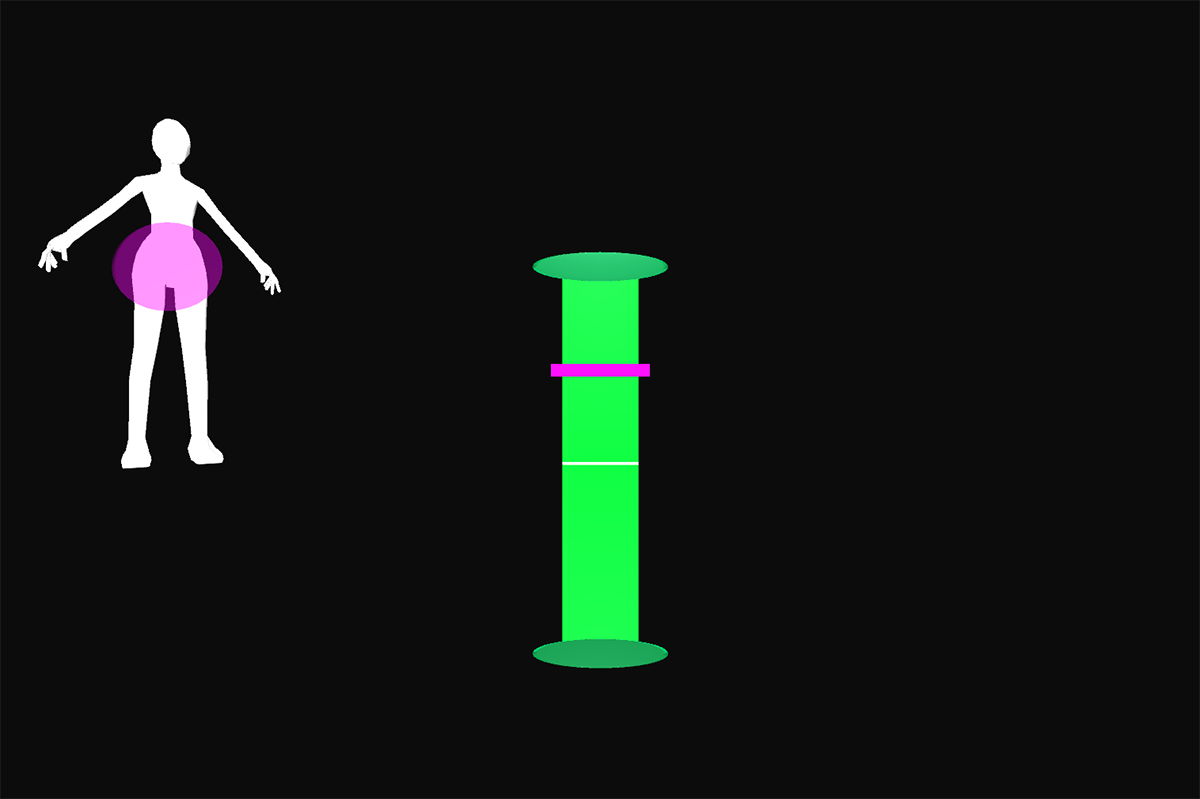

^a^Exercise 1: feedback visualization exercise 1.

### Data Processing and Analysis

Data were obtained via scribing logs and recordings of the thinking aloud process during the prototype testing and also using audio-recorded semistructured interviews after the prototype testing. The interview guide was not pilot-tested. The interview guide and the self-reported technical affinity questionnaire can be found in Multimedia Appendix 4. Audio recordings of the semistructured interviews were transcribed in a 2-step process, first by using the Happy Scribe Academic Research Transcription Service (Happy Scribe Ltd) and second, by revising the automatically created transcript executed by 2 members of the research team.

Qualitative content analysis (content structuring approach) according to Rädiker and Kuckartz [29] was conducted using MAXQDA Plus 2020 Release 20.1.0 (VERBI Software Consult Sozialforschung Gmbh). Data were transcribed, collected, coded, and reviewed using MAXQDA Plus 2020 Release 20.1.0 by 2 members of the research team. Interview and scribing data were merged and analyzed descriptively, forming the analysis unit. The coding categories were formed using a deductive-inductive approach. In the first step, categories were deductively derived from semistructured interview guide questions. In the second step, categories were formed inductively by summarization during the content analysis. Deductive and inductive subcategories were assigned to the analysis unit and single sentences, for example, single statements served as coding units.

**Figure 2:**
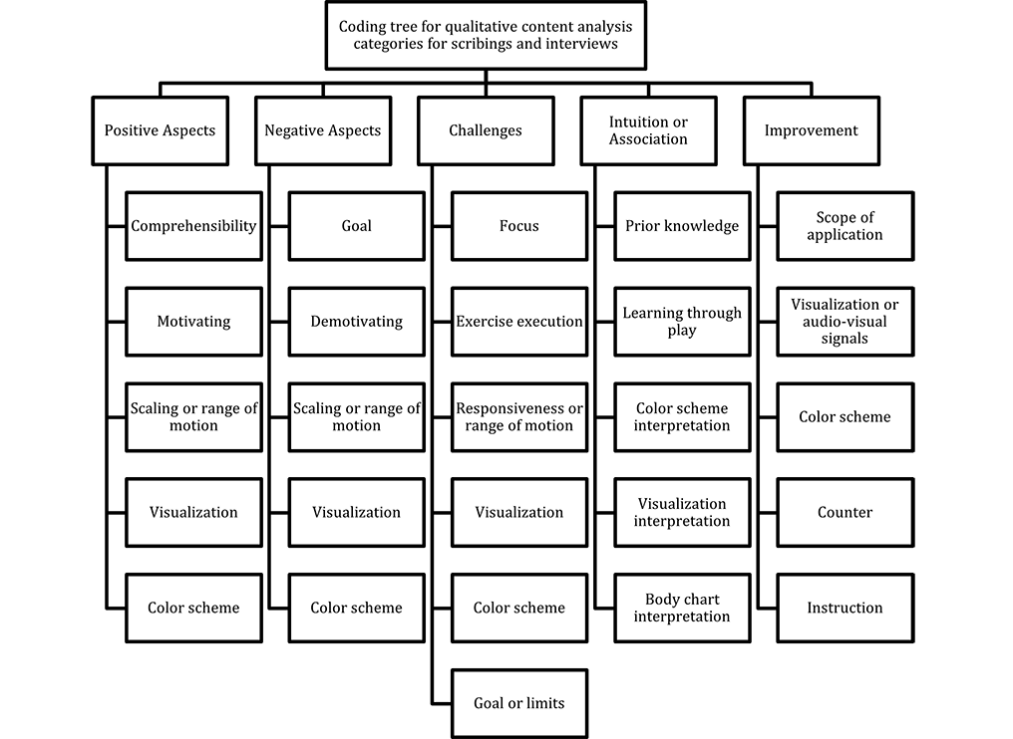
Deductive main categories and inductive subcategories used for qualitative content analysis visualized as a coding tree.

For the qualitative content analysis, 5 deductive main categories were used, directly extracted from the interview guide. The main category “positive aspects” was derived from the interview question, “What were the positive aspects of the feedback visualization?” The main category “negative aspects” stemmed from the question, “What were the negative aspects of the feedback visualization?” The main category “challenges” was based on the question, “What was the biggest challenge (while testing the real-time feedback)?” The main category “intuition/associations” was derived from the question, “How did you feel when you tried out the real-time feedback?” The main category “improvement” originated from the question, “Where do you see room for improvement in the feedback?” Five to six subcategories per main category were formed inductively during the content analysis, resulting in a total of 31 categories, comprising 5 deductive main categories and 26 inductive subcategories. An overview of the categories formed, presented as a coding tree, is shown in Figure 2.

To ensure the reliability of the coding process, the execution and analysis were conducted by 2 researchers with a professional and educational background in physiotherapy. The results and interpretations were reflected and discussed within the research team. Cross-tabulations were used to reassign interview and scribe log data to the different feedback visualizations.

### Researcher Characteristics and Reflexivity

All researchers involved in conducting the research (CK, KW, LM, and SD) have at least a master’s degree in health sciences, engineering, or both. All researchers were employees of FHCW. At the time of study execution, SD was also a part-time staff member at the participating hospital, OSS. CK identifies herself as female, and KW, LM, and SD identify themselves as male. KW was responsible for the study as principal investigator. The interview guide was developed by CK in collaboration with KW, both experienced physiotherapists with professional backgrounds in biomechanics and movement analysis. CK conducted the semistructured interviews and guided the participants throughout the prototype testing. KW carried out the scribing logs during prototype testing. SD was responsible for overseeing the laboratory equipment during prototype testing. CK and LM were responsible for transcription. LM and KW were responsible for data analysis using MAXQDA. This approach was chosen to enable researcher triangulation. Results were discussed among all participating researchers to enable peer debriefing, and therefore, reduce possible bias [30].

## Results

### Overview

A total of 10 participants were recruited, with 1 (10%) participant dropping out for personal reasons before prototype testing. Of the remaining 9 participants, 5 (56%) were physiotherapists, 2 (22%) were patients, and 2 (22%) were physicians; 89% (8/9) identified themselves as female and 11% (1/9) as male. Physiotherapists and physicians were either FHCW staff (3/9, 34%), OSS staff (2/9, 22%), or working in an unaffiliated interdisciplinary private practice (2/9, 22%). Participating patients were FHCW staff (2/9, 22%). None of the researchers had a private or patient-therapist relationship with the participants. Prior relationships were solely based on professional relations due to the same place of employment, either at FHCW, OSS, or private practice. Both participating patients were recruited based on personal contact, as the participants used publicly available contact information to contact the research team for study participation. Both patients reported osteoarthritis-associated functional deficits in the lower extremity due to prior traumatic knee injuries which were either a meniscal tear, ACL rupture in combination with a meniscal tear, or patellar tendon rupture. One (50%) patient described frequent pain episodes, and therefore, frequently used pain medication and 1 (50%) patient described pain after load, for example, running and did not report the use of any pain medication. Both patients were already undergoing regular prescribed physiotherapy. The mean age of the participants was 45 (SD 9) years. The mean work experience within relevant clinical fields among participating physiotherapists and physicians was 22 (SD 5) years. Concerning technical affinity, all participants used several technical devices daily, such as PCs, tablets, or smartphones. Only 3 (34%) of the 9 participants had no prior contact with technology in the context of therapeutic processes. These experiences ranged from exercise therapy videos over special anatomical software applications to the use of gait laboratory equipment or exoskeletons. Of the 9 participants, 4 (44%) participants stated high interest, 4 (44%) had medium interest, and 1 (11%) participant had low interest in technology in the context of therapeutic processes. Concerning the self-reported ability to learn about new technologies, 2 (22%) participants stated that they had very good ability, 6 (67%) stated that they had a good ability, and 1 (11%) stated that they had rather poor ability. The demographic characteristics of the participants are summarized in Table 2.

The self-reported technical affinity of the participants is summarized in Table 3.

**Table 2:** Demographic characteristics of study participants (N=9).

ID	Sex	Age^a^ (y)	Participant group	Years of work experience
FB01^b^	Female	44	Physiotherapist	20
FB02	Female	38	Patient	—^c^
FB03	Female	51	Physician	24
FB04	Female	37	Patient	—
FB05	Female	54	Physician	29
FB06	Female	59	Physiotherapist	24
FB07	Female	52	Physiotherapist	29
FB08	Male	30	Physiotherapist	6
FB09	Female	43	Physiotherapist	22

^a^Mean age was 45 (SD 9) years.

^b^FB01: feedback participant 1.

^c^Not available.

**Table 3:** Self-reported technical affinity of study participants.

ID	Daily use of technical devices	Number of apps installed on smartphone	Experience with technologies in the context of therapy	Interest in modern technology in the context of therapy	Ability to learn about new technologies
FB01^a^	Smartphone, tablet, PC, smartwatch	>5	Inclinometer on smartphone, camera for therapy-videos	High interest	Good
FB02	Smartphone, tablet, PC, smartwatch	>5	Continuous passive movement brace	High interest	Very good
FB03	Smartphone, PC, bicycle computer	>5	Exoskeleton	Medium interest	Very good
FB04	Smartphone, tablet, PC	>5	No experience	High interest	Good
FB05	Smartphone, tablet, smartwatch, smart home applications	>5	Gait and movement laboratory	Medium interest	Good
FB06	Smartphone, tablet, PC, game console	>5	No experience	Low interest	Rather poor
FB07	Smartphone, tablet, PC, smartwatch	>5	No experience	High interest	Good
FB08	Smartphone, PC	>5	Anatomy—software	Medium interest	Good
FB09	Smartphone, tablet, music player	>5	Therapy-exercise videos	Medium interest	Good

^a^FB01: feedback participant 1.

### Main Findings of Qualitative Content Analysis

#### Positive Aspects

This category summarizes all positive comments from participants regarding the feedback visualizations. Participants described the feedback visualizations as clear and self-explanatory. They mentioned that although a short period of familiarization was required, the visualizations became understandable and easy to control thereafter. The body chart effectively highlighted the body parts on which the feedback was provided. However, some participants noted that it took several attempts to accurately link the highlights within the body chart to the corresponding feedback visualization:

But the feedback was very, very positive. It was also very, very clear for most things...

Interview transcript FB08: 124

As if I look into a mirror and don’t have to rethink, target is symmetrical distance sun to blue bars.

Thinking aloud scribing FB05: 22

Motivational aspects, such as the direct exercise feedback, were perceived as playful. Participants highlighted that the additional external focus provided by real-time feedback visualizations was motivating and encouraged performing exercises with heightened awareness. In addition, they noted that real-time feedback visualizations would be applicable in therapy settings, particularly the visual control of body alignment. Positive feedback visualizations (exercises 6.1 and 6.2) were identified as the most motivating:

I think it is also cool for me as a therapist, if the patient is always evading with the upper body or with the pelvis, but otherwise it looks good, then I set the device exactly to the pelvis, so that I know, okay, now he knows, he must look at the pelvis and I can check.

Interview transcript FB08: 128

So, this exercise execution motivation I think it is great. Because the other feedback always tells me what I’m doing wrong.

Interview transcript FB01: 102

The feedback on the range of motion was generally perceived positively. Participants noted that the required range of deviation to trigger warning signs for better body axis control was within realistic boundaries with a participant stating, “In a realistic range, neatly dodge to reach forbidden zone” [Thinking aloud scribing FB01: 58].

The simple design of the feedback visualizations were considered beneficial in a therapy setting as they were pleasantly visible and appropriately sized. Highlighting 1 part of the body as a single feedback visualization was seen as a positive aspect for maintaining focus on exercise execution. In addition, visual cues, such as using the same color for highlights in the body chart and the pointer within the feedback visualization, were noted to enhance comprehensibility:

And at the beginning you also said, that’s a simple representation, would you also add that as a positive aspect?

Interviewer

Actually, yes.

FB05

And actually, it should be simple. It shouldn’t be that the patients say, “ah that’s complicated.”

Interview transcript FB05: 58 to 60

I thought it was somehow...more pleasant, when the bar had the same color as this oval circle on the body chart.

Interview transcript FB08: 60

Concerning the color used for representing feedback visualizations, participants mentioned, that they regarded traffic-light colored feedback visualizations using green-yellow-red as useful. Some mentioned, that staying within a green zone during the exercises conveyed a sense of security. They said, “...again, a new visualization, with a green, yellow, and red area, I like it” [Thinking aloud scribing FB08: 78]

#### Negative Aspects

In this category, all comments from participants that could be interpreted as negative regarding the feedback visualizations were summarized. Participants particularly viewed the lack of additional information negatively. For example, the feedback visualizations did not provide the number of repetitions or previous information concerning the exercise feedback. Due to the absence of repetition counters, participants felt they did not know the final goal of the exercise, which led to demotivation. One participant stated, “That you don’t know how many times to do it now [laughs]. What are you looking for?” [Interview transcript FB09: 96].

In addition, the boundaries for warning signs related to the range of motion were often considered too wide, necessitating extensive evasive movements. One participant stated:

It only reaches the red zone when you almost fall over…My patients don’t sway that much with their upper body that you could see such deviations.

Thinking aloud scribing FB07: 30

Regarding the illustration of feedback visualizations, participants mentioned that exercise 6.2 was confusing in terms of spatial perspective, as it was unclear whether the feedback was displayed in 2D or 3D. In addition, participants were irritated by pop-up notifications during the exercises, as they were not immediately understandable. It was also criticized that the body chart, which served as guidance throughout the exercise feedback, had no additional purpose other than highlighting the body region that received feedback:

Circle above irritated as it seems to be three dimensional, but I know it is displayed two dimensional.

Thinking aloud scribing FB01: 112

Hold the pelvis still. Yes. That irritated me.

Interview transcript FB02: 8

The color schemes of the feedback visualizations were criticized for their simplicity, as they might not be motivating or engaging enough for participants to stay focused during the exercise. In addition, the color combination of green and pink for the feedback visualizations and highlights within the body chart was deemed lacking in variation. Red was often viewed negatively due to its use as a warning symbol within the feedback visualizations. The variation of green tones in feedback exercises 6.1 and 6.2 was rated as unclear in describing the goal orientation of the feedback:

In our fast-paced world with many images and color impressions, I can imagine that it becomes a bit monotonous with time. That’s not a good thing, but we are unfortunately... live in a world where so many images, so many impressions come at us that we are not used to concentrate on three colors and three bars, or rather one bar and accept that there is not much more beyond that.

Interview transcript FB05: 4

#### Challenges

In this category, all comments from participants regarding the feedback visualizations, which could be associated with challenges while testing the system, were summarized. Especially during the initial feedback exercises, participants mentioned confusion about connecting the feedback visualization to their actual movement. Recognizing the body chart and its highlighted parts as indicators of which body region was receiving feedback within the visualization, was challenging for the participants. This sometimes led to them ignoring the feedback altogether:

... if I remember correctly, I was a bit irritated by what I saw and what I thought I wanted to do.

Interview transcript FB09: 84

The first time it was still a bit awkward. What does it pay attention to now? What am I doing? Where am I looking at?

Interview transcript FB08: 4

The execution of exercises in conjunction with real-time feedback visualization presented several challenges for the participants. Maintaining the leg axis alignment was particularly difficult, even with the assistance of the feedback. In addition, several participants reported using compensatory movements to keep the feedback in alignment. One participant aptly described this as “trying not to cheat”:

If I cause an evasive hip movement, it still looks like a good squat, as if my knee would stay straight, more attention needs to be paid to the pelvis.

Thinking aloud scribing FB08: 64

That you try not to cheat.

Interview transcript FB02: 12

Another challenge during exercise execution was related to the sensitivity and calibration of the system. Participants noted that if they changed their starting position during repetitive exercise execution, the system did not respond immediately. Consequently, their perception of properly aligned exercise execution differed from the displayed feedback visualization. For several exercises, participants observed that the feedback reaction was too sensitive. One participant even mentioned feeling distressed due to the fast and sensitive response while performing a dynamic exercise:

When I think I need to keep the bar in the middle, it feels like I need to swerve.

Thinking aloud scribing FB08: 75

I imagine it to be difficult if I place the foot somewhere else, because then the clearance changes.

Thinking aloud scribing FB08: 25

The exercise with visualization stresses more, I can make an effort, but it is difficult to execute, but this is perhaps due to the dynamics of the exercise.

Thinking aloud scribing FB05: 50

Interpreting the body chart, which highlighted the body region that received feedback, was initially challenging for participants. Several participants found it difficult to understand the meaning of the digital body chart, leading some to partially or completely ignore it:

At the beginning, I somehow didn’t consciously notice it. I just saw that there was a body chart, but I didn’t notice that the focus was somehow on it. And then I thought, no, I don’t want to consciously look at it now and somehow tried to find out where it is on my own.

Interview transcript FB07: 76

Another major challenge for participants was understanding the feedback itself without any additional information. Participants could not immediately distinguish the movement boundaries and often had to rely on trial and error. This was particularly noted for the last 2 feedback exercises (exercise 6.1 or 6.2), which required a specific range of motion, unlike the previous exercises (exercises 1-5.2). In addition, it was sometimes unclear not only how to perform an exercise but also how often it should be performed:

But it was not quite clear to me, what does the system want now just with some things? Should I now deliberately try to stay in this area, or should I deliberately try to reach larger areas?

Interview transcript FB08: 12

Yes, so at the beginning it wasn’t clear to me sometimes, for example, how long you want me to do some things. So, is there a certain number of repetitions where you say this is how often I must do it.

Interview transcript FB09: 4

#### Intuition or Association

In this category, all the participant’s comments related to feelings, intuitive interpretations, actions taken during testing, and associations with past therapeutic experiences were summarized. Some participants noted that their physiotherapeutic knowledge, whether from professional training or experience gathered during therapy, influenced their interpretation of the feedback visualizations. For example, this led them to avoid trying to reach the boundaries for the range of motion feedback, instead staying within a movement range that felt safe. One of them said, *“*That is, probably that was my premonition already, that I think to myself, okay, I should stay within a certain movement limit and just not do a far-reaching range of motion” [Interview transcript FB09: 56].

Participants expressed that they enjoyed the playful aspects of the feedback visualizations. Specifically, the final 2 feedback visualizations (exercise 6.1 or 6.2) were considered motivating due to their incorporation of a reward system for achieving a certain range of motion. In addition, other feedback visualizations were associated with a serious gaming approach. One visualization used for 2 exercises was particularly interpreted as resembling a tennis video game (exercise 2.1 or 4.1). The participant stated, “Reminds me of the first video game I had, tennis ball with bars back and forth.” [Thinking aloud scribing FB06: 79].

Participants described the color scheme used in the various feedback visualizations (green, yellow, and red) as intuitive and relatable. They associated these colors with a traffic light system, which they found logical and easy to understand. Consequently, red was intuitively interpreted as indicating an unwanted range of motion, yellow as a caution zone, and green as the desired range of motion:

The others were relatively logical in terms of their structure. Because you saw right away, okay, this is turning red, I definitely don’t want to go there.

Interview transcript FB08: 16

Green is range, yellow suboptimal, red attention/negative.

Thinking aloud scribing FB01: 45

Using different shades of green for positive feedback was not intuitively clear for all participants. As a result, they did not immediately recognize the goal of the feedback visualization in exercises 6.1 and 6.2:

Because for green by itself, I would associate it with the area of the best rated movement. So that’s the area where I should be. I didn’t get an assignment and that’s what I’ve assumed.

Interview transcript FB05: 64

As previously mentioned, the interpretation of feedback visualizations was not initially clear to all participants. Several participants used a trial-and-error approach to identify which body region was receiving feedback. Nonetheless, some participants were able to link the highlights on the body chart with the intended body region, allowing them to intuitively understand the feedback visualizations. Participants described the body chart differently; some interpreted it as a female character, others as diverse, and some could not distinguish whether it was depicted from the front or back:

Lady left remains the same with focus on knee.

Thinking aloud scribing FB05: 24

Still the figure with the knee, Ping-Pong ball, ah this is the right front knee.

Thinking aloud scribing FB02: 17

The figure—pelvis looks as if it were buttocks, looks as if it had a chest, remains static, wants to point me only toward the body region knee.

Thinking aloud scribing FB01: 10

#### Improvement

All comments by participants related to ideas for improving the feedback visualizations or expanding the field of application were summarized in this category. Concerning the extension and applicability of the feedback visualizations, participants presented several ideas. They emphasized the potential use of feedback systems for young people due to the playful approach of the feedback visualizations. In addition, participants suggested incorporating more elements of gamification, such as repetition counters, positive feedback through auditory signals, and reward systems. They also emphasized the importance of using progression models to adjust exercise difficulty. Furthermore, it was suggested that the feedback visualizations could be used in sports, as well as for health promotion and prevention:

... wrapping that up in a story or in a game.

Interview transcript FB05: 20

I would extend this and not only use it in the field of rehabilitation, but also in the field of athletics.

Interview transcript FB03: 56

It can prevent injuries—if you have good coordination in the first place and everyone can practice this at home very simply. So also, in the field of prevention. Especially for, old people who have poor balance. You can put together exercises for them to do at home to maybe prevent falls.

Interview transcript FB03: 60

The implementation of audio in addition to visualizing feedback was particularly emphasized by participants. They suggested using audio for verbal instructions of the exercises and for amplifying both positive and negative feedback. In addition, participants mentioned that explanatory videos before performing the exercises would be helpful for immediately understanding the purpose and execution of the exercises in conjunction with the feedback visualizations. A participant stated, “You could add an acoustic signal to it” [Interview transcript FB06: 52].

Regarding the color scheme, it was suggested that improvements could be made by incorporating orange as an additional color and using a fading, smooth transition between different colors within the feedback visualizations. Furthermore, the use of certain colors, such as red for negative feedback, was criticized by some participants. They suggested focusing on positive feedback and reward systems to enhance motivation and motor learning. Some colors, particularly the mixture of violet and red and the use of garish colors, were unpleasant for some participants. The participants stated that the use of different shades of green should be optimized, as the differentiation was unclear to most participants:

And there was a very wide yellow range, but I would probably say yellow and orange and red, so that red doesn’t lose its sharpness.

Interview transcript FB09: 40

...the combination of this purple and the red outside, that was somehow...unpleasant.

Interview transcript FB07: 40

...what I noticed; it is a bit deficit oriented as I would interpret it. Maybe it’s because I’m associating danger or “better off” with red, and the green area, maybe that staying in the green area should already be a reward. There might be potential for improvement.

Interview transcript FB06: 72

Implementing a counter, either for repetitions or for exercise duration, was frequently suggested. In addition, participants mentioned that statistical analysis in terms of terminal feedback or knowledge of result [28] after completing the exercise program could be beneficial:

So, for example, approximately a certain number of repetitions...

Interview transcript FB09: 100

I’m sure that if the device gave me a countdown, I would be highly motivated to finish it completely, while if I did it without...I just leave out two movements or something.

Interview transcript FB01: 46

Finally, participants noted that they needed more instruction and guidance while testing the feedback visualizations. This could be provided through visual instructions using videos or animations, audio elements explaining the exercises and the purpose of the feedback visualizations, or through descriptive elements:

You receive relatively little information at the beginning...With a little more information about what you should pay attention to, you could put more emphasis on the correct execution right from the start.

Interview transcript FB03: 12

### Summary of the Main Analytic Findings

Participants provided diverse feedback on the prototype feedback visualizations, emphasizing both positive and negative aspects as well as various challenges encountered during testing. In summary, while participants appreciated the clarity and motivational aspects of the feedback visualizations, they also highlighted areas for improvement, particularly regarding information clarity, color scheme effectiveness, and system responsiveness. To provide an overview of the main analytic findings, Table 4 lists the key findings within the corresponding main and subcategories.

**Table 4:** Main analytic findings per deductive main- and inductive subcategories concerning feedback visualization.

Deductive main category and inductive subcategories		Main analytic findings
**Positive aspects**		
	Comprehensibility	Feedback visualizations were described as clear and self-explanatory once familiarized.
	Motivating	Motivational aspects were highlighted, particularly the real-time feedback enhancing exercise engagement.
	Scaling or range of motion	The range of motion feedback was generally perceived positively.
	Visualization	Participants appreciated the simplicity and visibility of the feedback visualizations.
	Color scheme	The color scheme using green-yellow-red was intuitive and perceived as useful.
**Negative aspects**		
	Goal	Lack of additional information in the feedback visualizations was a major concern.
	Demotivating	Simplistic color schemes and lack of variation were criticized for potentially reducing motivation.
	Scaling or range of motion	Challenges with sensitivity and calibration of the system during exercise execution were highlighted. Some participants found the range boundaries too wide, and therefore misleading.
	Visualization	Issues with distinguishing 2D vs 3D feedback visualizations were noted.
	Color scheme	Simplistic color schemes and lack of variation were criticized for potentially reducing motivation.
**Challenges**		
	Focus	Initial confusion and difficulty in connecting feedback visualizations to actual movements were present.
	Exercise execution	Challenges in maintaining alignment and avoiding compensatory movements during exercises were noted.
	Responsiveness and range of motion	The sensitivity and responsiveness of the feedback posed challenges during dynamic exercises.
	Visualization	Participants struggled with interpreting the body chart and its relevance.
	Color scheme	Interpreting low-contrast color schemes (shades of green) was challenging and unclear.
	Goal or limits	Understanding exercise goals was often unclear.
**Intuition or association**		
	Prior knowledge	Professional knowledge and intuitive interpretations influenced participants’ interactions with the feedback.
	Learning through play	Enjoyment of the playful aspects of certain feedback visualizations was expressed.
	Color scheme interpretation	Participants associated the traffic-light color scheme with intuitive meanings.
	Visualization interpretation	Interpretation of feedback visualization was unclear initially; participants used trial-and-error to identify body parts receiving feedback.
	Body chart interpretation	Participants had varied interpretations of the body chart and some could not tell if it was shown from the front or back.
**Improvement**		
	Scope of application	Participants proposed extending the system’s use beyond rehabilitation to include other use cases, such as sports.
	Visualization or audio-visual signals	Recommendations included integrating audio feedback and implementing gamification elements.
	Color scheme	Suggestions for improving the color scheme (including orange, using nonprejudgmental colors) and feedback clarity were common.
	Counter	Including elements like repetition counters and enhancing feedback with more detailed performance statistics was proposed.
	Instruction	Enhancing exercise instructions and using instructional videos was suggested.

## Discussion

### Principal Findings

Within our qualitative study examining usability factors of prototype feedback visualizations for real-time exercise feedback, participants mostly reported high to medium interest in technology in the context of therapy. Overall, participants enjoyed the prototype feedback visualizations, and visualizations were considered simple, clear, and self-explanatory. The main criticism was the lack of additional information and missing additional color schemes to further highlight and distinguish movement deviations. Advice for improvements, such as increasing the use of positive feedback and incorporating additional gamification elements, was given by participants. No one dropped out during the prototype testing and no adverse events occurred during the testing.

### Comparison With Previous Studies

Participants were positive about the simple, clear, and self-explanatory visualizations; however, they stated that a short period of familiarization was needed. In addition, they believed that the feedback prototype could be well applicable in a therapy setting. Ling et al [31] report similar results for patients who tested their system as they emphasize the usefulness of an exergame for rehabilitation purposes. Another positive remark of participants was toward the simple design and the large size of the feedback visualization. In addition, the use of a traffic-light color scheme for the feedback visualization was viewed positively. Staying in the green zone during an exercise gave the participants a feeling of safety and visual cues were also regarded as helpful. Those improvements were incorporated based on learnings after the first prototype iterations presented by Widhalm et al [26]. These feedback visualizations correspond with findings of Sun et al [32], who stated, that people enjoy simple and common designs to reduce their mental load.

Nevertheless, negative aspects were reported regarding the prototype feedback visualizations. A main point of criticism was the lack of additional information given by the system during the prototype testing. It was intentional to test whether the design of the feedback visualizations is self-explanatory or not. Participants were invited to explore the feedback modalities and the mechanics of the systems on their own but under supervision, which led to positive failure and learning. This approach is also described in the study by Lohse et al [33]. The participants criticized that no additional information regarding the number of repetitions and no previous information concerning the exercise-feedback were given in advance of the exercises by the system itself. This led to challenges in exercise execution and partial misinterpretation of prototype feedback visualizations. Some participants mentioned that this was especially demotivating. The importance of clear goal setting and instructions is emphasized by Lohse et al [33], as a lack of goal-directed tasks can lead to reduced motivation and acceptance. This is in line with the suggestions for possible improvements of the system as pointed out by participants, who mentioned that introductory elements would be a beneficial optimization. This could either be delivered by visual instructions with the help of videos or animations, with audio elements explaining exercises and the purpose of the feedback visualization, or with descriptive elements. Incorporating a counter, either for repetitions or for exercise duration, was frequently mentioned by participants. The importance of tracking metrics, such as repetition counting is also reported in Ananthanarayan et al [34] to, for example, give the possibility to trace improvements over time. In addition, statistical analysis after performing a set of exercises could be beneficial for additional motivation and to emphasize the frequent use of the system to further improve motor learning. Different methods of incorporating game design elements in an exergame scenario, such as the presented real-time feedback visualization, are reported in Martinho et al [35]. It is proposed that with the use of design elements for tracing improvements, participants may be motivated to further improve their abilities [35].

Feedback on chosen color schemes highlighted similar implications for optimization. Participants mentioned that additional colors could be implemented in the traffic light system, such as orange, to be able to further distinguish the movement deviation within the feedback visualizations. Also, a fading between different colors used within the feedback visualizations could be helpful. Nonetheless, the simplicity of the colors used was partly criticized. Participants were unclear concerning the goal of the overall green feedback visualizations exercises 6.1 and 6.2. Using red as a warning signal was also criticized by some participants. This is an important learning in terms of improving the color schemes and used effects within the feedback visualizations, as negative feedback can lead to a negative emotional response by participants, which is also highlighted in a study by Ravaja et al [36]. They describe in their study on emotional reactions to video games that negative emotions especially arose, when participants received a replay of a failure, and therefore, were not actively involved anymore in the video game itself [36]. Therefore, hypothetically, a similar effect could have led to negative associations of the feedback visualizations in this study, as misinterpretation or unclear goals of the feedback visualizations could have led to a feeling of passive involvement and not being in control of the situation resulting in negative emotions.

The participants reported several challenges concerning feedback visualizations. The main challenge for participants was coupling the feedback visualizations with their actual movement and building a connection between exercise, body chart visualization, feedback visualization, and body regions that received feedback. This challenge resulted in partly ignoring the feedback visualizations overall and focusing only on themselves. The lack of interactivity due to not being able to couple the movements with the feedback visualizations is another improvable factor, as a well-elaborated interactivity can help participants to reach a feeling of ownership toward the digital real-time feedback system, and therefore, increase motivation [33]. Exercise execution by itself in conjunction with the feedback visualization was challenging for participants. Along with those difficulties, some participants described discomfort with the high sensibility and calibration of the system when, for example, changing the starting position during an exercise set. Therefore, the current system could be optimized in terms of dynamic difficulty adjustment, a theory described for video games by Hunicke [37], and integrated into serious game approaches, for example, in a speech therapy game by Martins and Cavaco [38]. Choosing a comfort policy according to Hunicke [37], which aims to be challenging but still manageable in terms of exercise difficulty and sensitivity of the feedback visualizations could help to prevent discomfort while using feedback systems during exercising. This could support participants reaching a flow state, a state of heightened awareness where the sense of time diminishes during exercising [39].

Incorporating further gamification elements and reward systems may be beneficial for additionally increasing motivation. The goal-oriented, reward-based feedback visualizations within the study were markedly described as positive in terms of being motivating as well as being a joyful approach to exercise therapy. For example, Mubin et al [40] state that it is crucial to establish a contextual framework of gameplay within serious games, ensuring that all player actions possess meaningful and pertinent significance and that the game is target-based to ensure a successful application.

### Limitations

Even though the study highlighted several important aspects in terms of prototype feedback visualizations, this study has several limitations.

First, the sample itself was recruited from FHCW networks, and therefore, may not be representative of a broader population. Participants were aged <60 years representing young and middle adulthood. In addition, most participants were already familiar with the use of technology in the context of rehabilitation. The sample size of 9 participants was implemented following the findings of Nielsen [25] for thinking aloud usability testing. It is also described elsewhere that 4 to 5 participants can identify up to 80% of usability issues in usability testing [41]. Therefore, for the subgroup physiotherapists (5/9, 56%), most of the usability factors may have been highlighted but for both subgroups patients (2/9, 22%) and physicians (2/9, 22%), a larger sample may have been beneficial and could have broadened the findings. Contributing to this, a possible limitation due to researcher as well as participant bias could have occurred. Nonprobability, purposive sampling was used and most of the participants were recruited from FHCW networks. Professional relationships due to similar work environments were present. Participant bias is a common problem in qualitative research, which can lead to the presentation of experiences in a way that participants believe conforms to the researcher’s expectations or social norms. Researcher bias unintentionally influences how data are perceived, and conclusions are drawn. We were aware of those possible biases in the design of the study. Hence, we thoroughly informed participants and obtained informed consent, built a nonjudgmental and open environment while prototype testing, and used researcher triangulation as well as research team discussions for peer debriefing during data analysis [30]. Thus, it cannot be guaranteed, that the discussed findings and the identified usability issues as well as possibilities for improvement are exhaustive and are generalizable beyond the study population. In addition, it must be noted, that the findings may differ in an old adulthood population.

Second, participants only tested the prototype feedback visualizations developed within the study’s corresponding research project for the homeSETT system and no other real-time feedback systems for exercise therapy. Therefore, the findings concerning the visualizations and feedback modalities may not be directly applicable for real-time exercise feedback on other developed projects.

Third, the feedback modality used was knowledge of performance. Even though knowledge of performance and knowledge of result as feedback modalities can have the same implication on performance, the learning strategies between those modalities differ [28]. Knowledge of performance enables participants to immediately receive feedback on the quality of their movement. Adding knowledge of result, for example, with additionally adding a counter for repetitions during a predefined time frame or a statistical analysis on how well each repetition was performed at the end of a set could further increase motivation and help the participants and therapists monitor improvements. In addition, only concurrent and no terminal feedback was used in this study. Incorporating different and more feedback modalities and giving terminal feedback to participants may lead to different results.

### Future Directions

Despite these limitations, participants testing the prototype feedback visualizations emphasized that it can positively influence motivation and also be applicable in other fields, such as sports or prevention. To the best of our knowledge, only a few other studies incorporated qualitative usability testing in the development of feedback systems [31,34,42], despite the necessity of combining evidence-based feedback interventions with qualitative user-centered design processes [43]. The further development of the homeSETT system for exercise therapy within the SETT research project will incorporate the findings of this study on usability factors for feedback visualizations and future research will follow the 2-fold process presented by Pirovano et al [44] for evaluation of the prototype system. This study presented a qualitative evaluation regarding the usability factors of the prototype on-screen feedback visualizations. Future research must highlight the applicability and validity of the therapeutic efficacy of the homeSETT system for exercise therapy within a clinical trial.

### Conclusions

This paper presents findings from a qualitative study on usability factors using the methods of thinking aloud, scribing logs, and semistructured interviews to assess user requirements for prototype on-screen feedback visualizations. The prototype feedback visualizations were perceived as positive by participants. Participants noted that the prototype feedback visualizations could be applied well in therapy settings. Overall, participants emphasized that the prototype feedback visualizations were simple, clear, and self-explanatory, but gave broad-reaching advice for optimizing the presented visualizations, such as incorporating additional game design elements for information purposes. Future work will integrate the gathered knowledge to optimize the prototype feedback visualizations and incorporate them in a further iteration of the homeSETT system. Future research must focus on the applicability and efficacy of the homeSETT system in the framework of a clinical trial.

## Appendix

Multimedia Appendix 1 Consolidated Criteria for Reporting Qualitative Research (COREQ) checklist.

Multimedia Appendix 2 Development of the iterative prototype feedback visualization protocol.

Multimedia Appendix 3 Video of the system setup and testing of the prototype visualization exercises 1 to 6.2 on the Gait Real-Time Analysis Laboratory system.

Multimedia Appendix 4 Interview guide and report form for self-reported technical affinity used in the study.

## Abbreviations

COREQ: Consolidated Criteria for Reporting Qualitative Research
FHCW: FH Campus Wien
GRAIL: Gait Real-Time Analysis Laboratory
OSS: Orthopedic Hospital Speising

## Data Availability

The datasets generated during and analyzed during this study are available from the corresponding author on reasonable request.
