# Supplementary material for: Real-Time Digitized Visual Feedback in Exercise Therapy for Lower Extremity Functional Deficits: Qualitative Study of Usability Factors During Prototype Testing
Source: JMIR Serious Games. 2024 Dec 10;12:e51771. doi: 10.2196/51771 (PMC11632893; doi:10.2196/51771)
Supplement: Multimedia Appendix 2 [file games_v12i1e51771_app2.pdf]

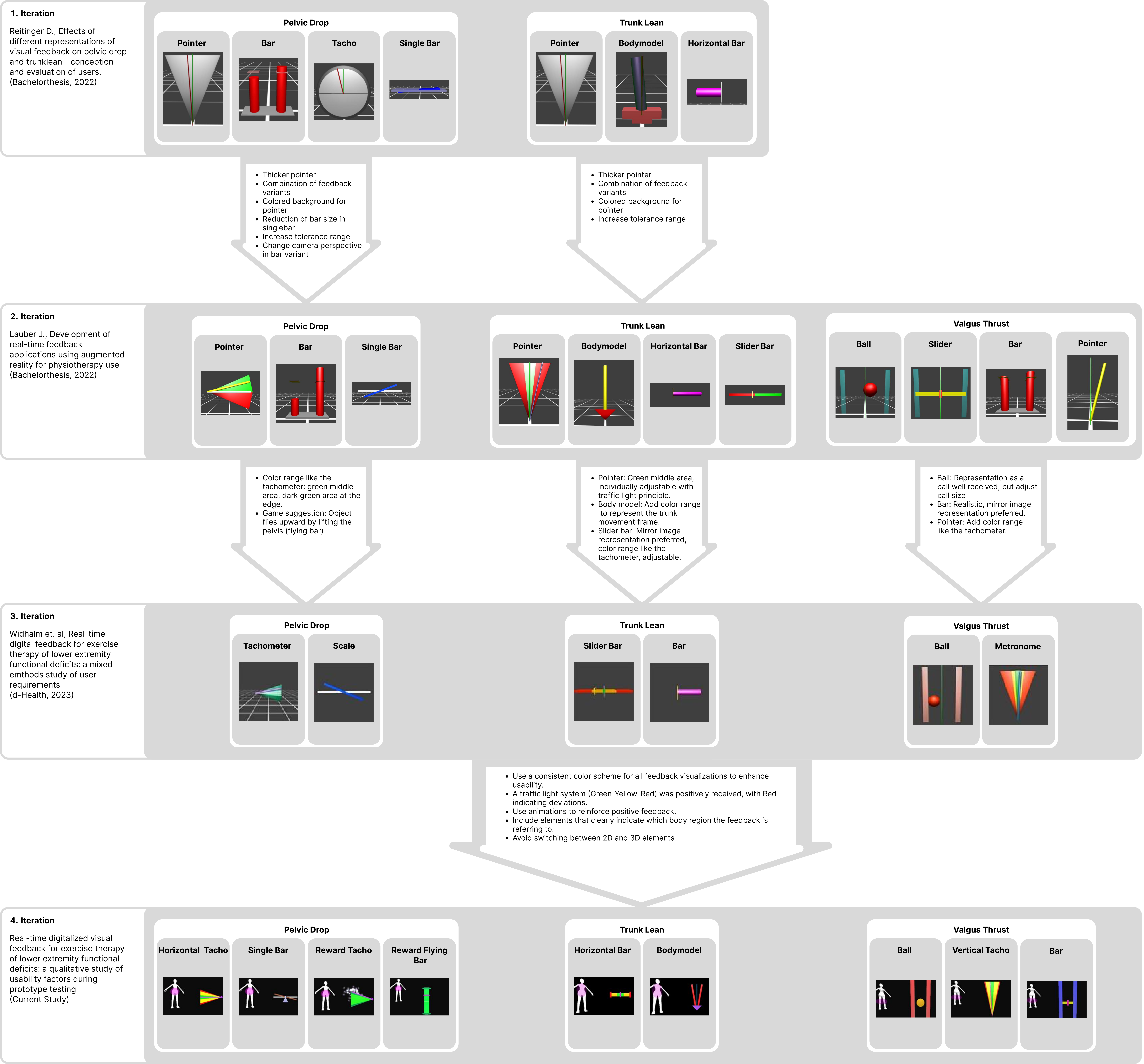
1. Iteration
Reitinger D., Effects of different representations of visual feedback on pelvic drop and trunklean - conception and evaluation of users. (Bachelorthesis, 2022)
Pelvic Drop
Pointer
Bar
Tacho
Single Bar
Trunk Lean
Pointer
Bodymodel
Horizontal Bar
• Thicker pointer
• Combination of feedback variants
• Colored background for pointer
• Reduction of bar size in singlebar
• Increase tolerance range
• Change camera perspective in bar variant
• Thicker pointer
• Combination of feedback variants
• Colored background for pointer
• Increase tolerance range
2. Iteration
Lauber J., Development of real-time feedback applications using augmented reality for physiotherapy use (Bachelorthesis, 2022)
Pelvic Drop
Pointer
Bar
Single Bar
Trunk Lean
Pointer
Bodymodel
Horizontal Bar
Slider Bar
Valgus Thrust
Ball
Slider
Bar
Pointer
• Color range like the tachometer: green middle area, dark green area at the edge.
• Game suggestion: Object flies upward by lifting the pelvis (flying bar)
• Pointer: Green middle area, individually adjustable with traffic light principle.
• Body model: Add color range to represent the trunk movement frame.
• Slider bar: Mirror image representation preferred, color range like the tachometer, adjustable.
• Ball: Representation as a ball well received, but adjust ball size
• Bar: Realistic, mirror image representation preferred.
• Pointer: Add color range like the tachometer.
3. Iteration
Widhalm et. al, Real-time digital feedback for exercise therapy of lower extremity functional deficits: a mixed emthods study of user requirements (d-Health, 2023)
Pelvic Drop
Tachometer
Scale
Trunk Lean
Slider Bar
Bar
Valgus Thrust
Ball
Metronome
• Use a consistent color scheme for all feedback visualizations to enhance usability.
• A traffic light system (Green-Yellow-Red) was positively received, with Red indicating deviations.
• Use animations to reinforce positive feedback.
• Include elements that clearly indicate which body region the feedback is referring to.
• Avoid switching between 2D and 3D elements
4. Iteration
Real-time digitalized visual feedback for exercise therapy of lower extremity functional deficits: a qualitative study of usability factors during prototype testing (Current Study)
Pelvic Drop
Horizontal Tacho
Single Bar
Reward Tacho
Reward Flying Bar
Trunk Lean
Horizontal Bar
Bodymodel
Valgus Thrust
Ball
Vertical Tacho
Bar
