# Supplementary material for: Real-Time Digitized Visual Feedback in Exercise Therapy for Lower Extremity Functional Deficits: Qualitative Study of Usability Factors During Prototype Testing
Source: JMIR Serious Games. 2024 Dec 10;12:e51771. doi: 10.2196/51771 (PMC11632893; doi:10.2196/51771)
Supplement: Multimedia Appendix 4 [file games_v12i1e51771_app4.pdf]

Supplementary File
Report form and interview guide

Participant: FB___ Date:

Age: _____

Gender: O m O f O d

Subtype: O Physiotherapist O Patient*in O other Stakeholder

If physiotherapist / other stakeholder:

Years of work experience: _______

Affinity for technology:

- **Which of the following devices do you use regularly in your everyday life?**

O Smartphone
O Tablet
O Laptop
O Smartwatch
O Other:

- **How many apps do you regularly use on your smartphone or tablet?**

O only phonecalls and text-messaging
O 3 to 5 apps
O more than 5 apps

- **Have you already gained experience with technology-assisted therapy?**

O Yes O No

If so, in what way:

____

____

- **How would you rate your interest in modern technologies?**

O no interest at all
O low interest
O medium interest
O high interest

- **How do you generally feel concerning your ability to learn about new technologies?**

O very good
O good
O rather poor
O bad

Qualitative interview guide

Main deductive category	Key question
Intuition / Association	How did you feel when you tried out the real-time feedback?
Challenges	What was the biggest challenge (while testing the real-time feedback)?
Positive aspects	What were the positive aspects of the feedback visualization?
Negative aspects	What were the negative aspects of feedback visualization?
Improvement	Where do you see room for improvement in the feedback?
Supportive question if applicable	At the beginning of exercise xy, you were puzzled by the feedback display. Why?
